# Ratio of low molecular weight serum adiponectin to the total adiponectin value is associated with type 2 diabetes through its relation to increasing insulin resistance

**DOI:** 10.1371/journal.pone.0192609

**Published:** 2018-03-01

**Authors:** Minoru Iwata, Kazuo Hara, Yutaka Kamura, Hisae Honoki, Shiho Fujisaka, Manabu Ishiki, Isao Usui, Kunimasa Yagi, Yasuo Fukushima, Atsuko Takano, Hiromi Kato, Shihou Murakami, Kiyohiro Higuchi, Chikaaki Kobashi, Kazuhito Fukuda, Yukiko Koshimizu, Kazuyuki Tobe

**Affiliations:** 1 First Department of Internal Medicine, Faculty of Medicine, University of Toyama, Toyama, Toyama, Japan; 2 Health Administration Center, University of Toyama, Toyama, Toyama, Japan; 3 Division of Endocrinology and Metabolism, Department of Comprehensive Medicine, Saitama Medical Center, Jichi Medical University, Omiya-ku, Saitama, Japan; 4 Department of Diabetology, Endocrinology and Metabolism, Tokyo Medical University, Shinjuku-ku, Tokyo, Japan; 5 Department of Internal Medicine, Asahi General Hospital, Asahi-machi, Toyama, Japan; 6 Division of Endocrinology and Metabolism, Department of Internal Medicine, Saiseikai Takaoka Hospital, Takaoka, Toyama, Japan; 7 Division of Endocrinology and Metabolism, Department of Internal Medicine, Japan Community Health care Organization Takaoka Fushiki Hospital, Takaoka, Toyama, Japan; 8 Division of Endocrinology and Metabolism, Department of Internal Medicine, Toyama Rosai Hospital, Uozu, Toyama, Japan; 9 Department of Internal Medicine, JA Niigata Kouseiren Itoigawa General Hospital, Itoigawa, Niigata, Japan; 10 Department of Internal Medicine, Kamiichi General Hospital, Kamiichi-machi, Toyama, Japan; 11 Department of Internal Medicine, Fukuda Clinic, Himi, Toyama, Japan; 12 Department of Internal Medicine, Urata Clinic, Kanazawa, Ishikawa, Japan; Tokyo Daigaku, JAPAN

## Abstract

**Aim:**

Among the three adiponectin isoforms, a lower ratio of high molecular weight (HMW) adiponectin to total adiponectin (TA) is well known to cause insulin resistance and type 2 diabetes (T2D). However, how the levels of other adiponectin isoforms, such as the middle molecular weight (MMW) and low molecular weight (LMW) isoforms, and their relative ratio to TA change in T2D subjects has not been determined. Therefore, we investigated the association of these adiponectin-related parameters with T2D.

**Methods:**

We examined the associations between adiponectin-related parameters and diabetes in a group of 394 T2D subjects and 374 controls (1^st^ group) randomly selected from among the participants in our previous study. The associations between these parameters and the HOMA-IR in a 2^nd^ group, consisting of the subjects remaining in the 1^st^ group after the exclusion of subjects receiving diabetic medication, were also examined.

**Result:**

In the 1^st^ group, after adjusting for confounding factor, the levels of all the adiponectin isoforms and the HMW/TA ratio were significantly lower among the diabetic subjects than among the controls (all *P* values < 0.01). On the contrary, the LMW/TA ratio was significantly higher among the diabetic subjects (*P* < 0.01) and was positively associated with T2D (odds ratio = 8.64, *P* < 0.01). In the 2^nd^ group, the HMW/TA ratio was inversely associated with the HOMA-IR; however, the LMW/TA ratio was positively associated with the HOMA-IR (β for LMW/TA ratio = 0.89, SE = 0.24, *P* < 0.001), similar to the association with T2D. The MMW/TA ratio was not associated with T2D or the HOMA-IR.

**Conclusion:**

The current investigation demonstrated that, unlike the reduction in the levels of all the adiponectin isoforms and the HMW/TA ratio, an increased LMW/TA ratio was associated with T2D through its relation to insulin resistance.

## Introduction

Adiponectin is mainly produced in adipocytes, although its production can sometimes occur or be up-regulated in other tissues such as the liver, skeletal muscle, and cardiac muscle, especially in the presence of inflammation[1–5]. This adipokine has been reported to ameliorate insulin sensitivity and to decrease the risk of type 2 diabetes (T2D) [6–10]. In addition, adiponectin consists of three isoforms: high molecular weight (HMW) oligomers, middle molecular weight (MMW) hexamers, and low molecular weight (LMW)trimers [11, 12]. Among these three isoforms, HMW adiponectin is the most biologically active isoform concerning insulin sensitivity, enabling predictions of the development of metabolic syndrome and T2D [13–15]. In addition, an association between a lower ratio of HMW/total adiponectin and T2D has been reported by Zhu et al. and another group [15, 16]. On the other hand, the roles of MMW and LMW adiponectin in the onset of diabetes are less well determined. In studies conducted among octogenarian Caucasians and among elderly individuals living in Japan, decreased LMW adiponectin levels were reportedly related with T2D [17, 18]. In contrast, in another study conducted among middle-aged Japanese-Brazilians, the levels of MMW and LMW adiponectin were similar between diabetic subjects and controls [19]. Thus, the roles of MMW and LMW adiponectin in the risk of diabetes remain controversial. In addition, some studies investigating other diseases have demonstrated that the ratios of these adiponectin isoforms to the total adiponectin level were useful biomarkers. For example, we previously reported that a high LMW/total adiponectin ratio and a low MMW/total adiponectin ratio were significantly related with current asthma [20]. Moreover, other studies reported that a high LMW/total adiponectin ratio was related with a decreased risk of Barrett’s esophagus [21]. However, to the best of our knowledge, the associations between T2D and the ratios of MMW and LMW adiponectin to the total adiponectin level have not yet been examined.

With the above-described background in mind, we enrolled a cohort consisting of diabetic subjects and non-diabetic control subjects and examined the associations of the levels of HMW, MMW and LMW adiponectin and their relative ratios to the total adiponectin level with diabetes and insulin resistance.

## Materials and methods

### Participants

**Fig 1:**
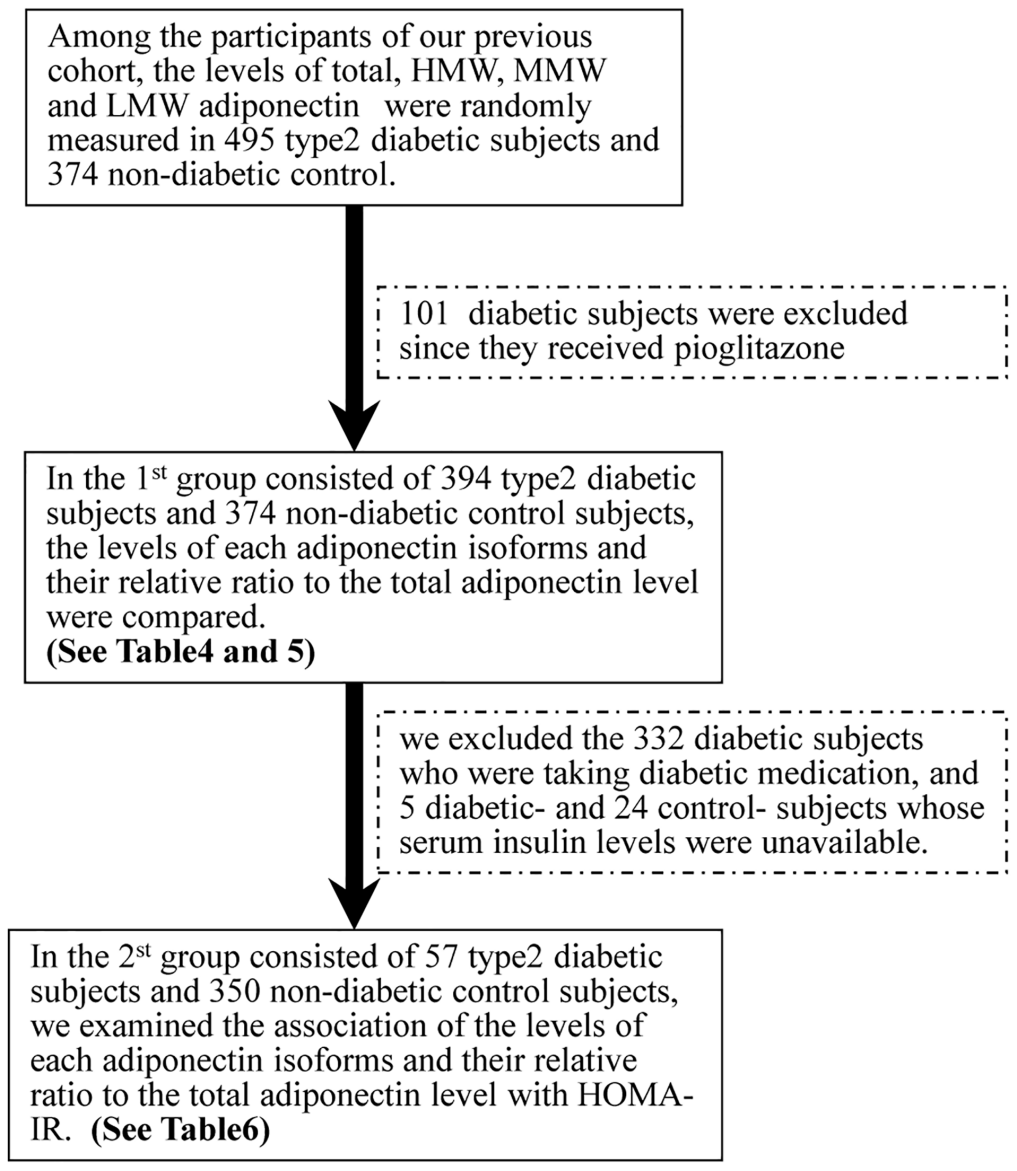
Selection of study subjects and analyses in the 1^st^ and 2^nd^ groups. HMW, high molecular weight; MMW, middle molecular weight; LMW, low molecular weight.

**Fig 2:**
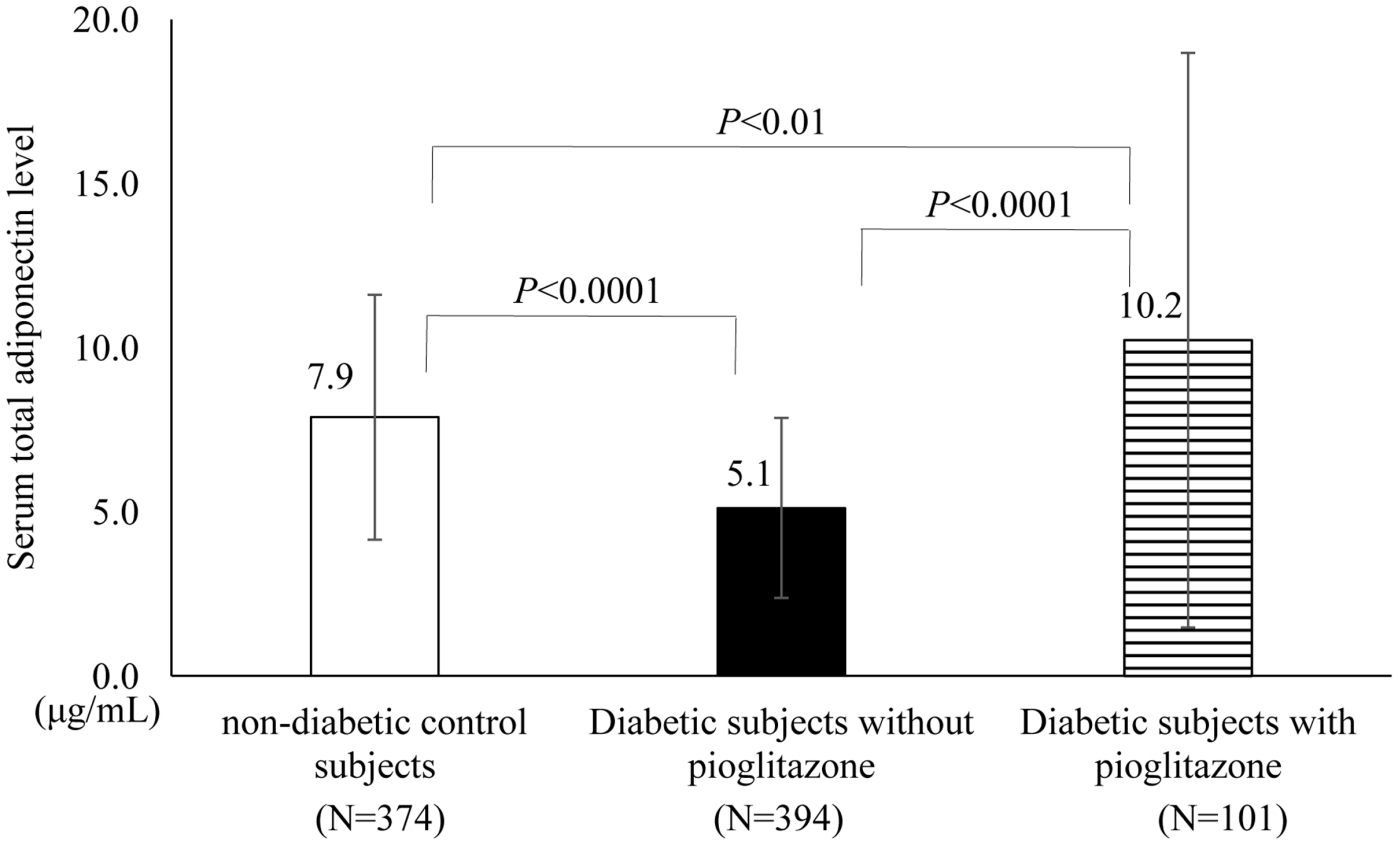
Comparison of the serum total adiponectin level between diabetic subjects treated with and without pioglitazone.

We enrolled a study cohort conducted between January 2008 and December 2009; this cohort consisted of 724 patients with T2D and 763 non-diabetic control individuals (CON) who had participated in a previously performed genome-wide association study examining genetic loci associated with type 2 diabetes in the Japanese population [22] (Fig 1). Among these participants, we randomly measured the levels of the three adiponectin isoforms in 495 T2D and 374 CON subjects (Fig 1). Since thiazolidinediones (TZDs) such as pioglitazone have been reported to increase the serum adiponectin level by about twofold (compared with levels in subjects who were not taking TZDs) [23] [24] and this finding was confirmed in the present study (Fig 2), we excluded 101 subjects with T2D who had been taking pioglitazone to minimize the influence of the diabetic medication on the serum adiponectin levels as much as possible (Fig 1). Finally, the levels of HMW, MMW and LMW adiponectin and their relative ratios to the total adiponectin level were compared between 394 T2D and 374 CON subjects (1^st^ group) (Fig 1). The clinical characteristics of the subjects in the 1^st^ group are shown in Table 1. Next, to evaluate the association of the above adiponectin-related parameters with the homeostasis model assessment of insulin resistance (HOMA-IR), which is an index of insulin resistance, subjects in the 1^st^ group who were taking diabetic medication were excluded, since agents such as sulfonylureas (SU) might affect the serum insulin level. In addition, we also excluded subjects whose serum insulin levels were unavailable (Fig 1). Finally, the above-mentioned associations were analyzed in 57 T2D and 350 CON subjects (2^nd^ group) (Fig 1). The clinical characteristics of the subjects in the 2^nd^ group are shown in Table 2. The inclusion criteria for non-diabetic controls and the exclusion criteria for the cases were described in our previous reports [22, 25]. Briefly, the inclusion criteria for non-diabetic controls were as follows: (1) >50 years of age, (2) HbA1c values <6.0%, and (3) no past history of a diagnosis of diabetes as previously described [22, 25]. Diabetes was diagnosed based on the 1998 American Diabetes Association Criteria [26]. The exclusion criteria for the cases with diabetes were individuals with diabetes caused by (1) liver dysfunction, (2) steroids and other drugs that might increase glucose levels, (3) malignancy, and (4) individuals who tested positive for anti-GAD antibody, as previously described [22, 25].

**Table 1:** Clinical profiles of the study subjects in the 1^st^ group.

	Type 2 diabetes	Control	*P* value
N	394	374	
Sex (M/F)	257/ 137	156/ 218	<0.0001*
Age (years)	63.9±10.9	67.4±8.0	<0.001
Duration of diabetes (years)	13.4±8.8	-	
Age at diagnosis (years)	50.5±11.8	-	
Self-reported family history of diabetes (%)	54.6	-	
BMI (kg/m^2^)	24.2±3.8	23.0±3.0	<0.0001
Waist circumference (cm) (male)	86.7±9.1	84.0±8.2	<0.01
Waist circumference (cm) (female)	86.6±11.2	81.9±9.0	<0.001
S-Cre (mg/dL)	0.80±0.35	0.75±0.18	0.602
FPG (mg/dl)	136.5±34.9	96.5±8.6	<0.0001
HbA1c (NGSP value) (%)	7.62±1.37	5.48±0.27	<0.0001
Use of antihypertensive agent (%)	55.6	36.1	<0.0001*
Use of lipid lowering drug (%)	46.4	21.9	<0.0001*
Diabetes therapy			
Diet alone (%)	15.7		
Using Oral hypoglycemic agents (%)	66	-	
Sulfonylureas (%)	42.1	-	
Biguanides (%)	27.9	-	
α-glucosidase inhibitor (%)	30.7	-	
Glinide (%)	4.6	-	
Using Insulin (%)	40.1	-	

Data are means ± SD.

* Pearson’s chi-square test.

BMI, body mass index; S-Cre, serum levels of creatinine; FPG, fasting plasma glucose

**Table 2:** Clinical profiles of the study subjects in the 2^nd^ group.

N	407
Sex (M/F)	187/ 220
Age (years)	66.9±8.5
BMI (kg/m^2^)	23.1±3.0
Waist circumference (cm) (male)	84.4±8.3
Waist circumference (cm) (female)	81.7±9.4
S-Cre (mg/dL)	0.75±0.18
FPG (mg/dl)	100.5±14.7
HbA1c (NGSP value) (%)	5.66±0.65
HOMA-IR	1.34±0.92
Use of antihypertensive agent (%)	37.9
Use of lipid lowering drug (%)	24.8
Total-Adiponectin (μg/mL)	7.40 ± 3.64
HMW-Adiponectin (μg/mL)	3.54 ± 1.73
MMW-Adiponectin (μg/mL)	1.61 ± 0.86
LMW-Adiponectin (μg/mL)	2.25 ± 1.06
HMW/Total	0.44 ± 0.13
MMW/Total	0.23 ± 0.07
LMW/Total	0.33 ± 0.11

Data are means ± SD.

BMI, body mass index; S-Cre, serum levels of creatinine; FPG, fasting plasma glucose; HOMA-IR, the homeostasis model assessment of insulin resistance; Total, total adiponectin; HMW, high molecular weight; MMW, middle molecular weight; LMW, low molecular weight

All the study procedures were approved by the Ethics Committee of the University of Toyama, and written informed consent was obtained from all the study subjects.

The serum total adiponectin level was logarithmically (naturally) transformed, and the differences in the levels between each group were examined using the Student *t*-test.

### Collection of clinical information

The anthropometric measurements of individuals wearing light clothing and without shoes were conducted by well-trained examiners. Body height and weight were measured to the nearest 0.1 cm and 0.1 kg, respectively. The BMI was calculated from these measurements. The waist circumference (WC) measurements were obtained at the end of normal expiration and were measured to the nearest 0.1 cm at the umbilical level using a flexible anthropometric tape. In addition, we obtained clinical information including the family history of diabetes, the age at diagnosis, and use of antidiabetic drugs, antihypertensive agents, and lipid lowering drugs from medical records and self-reported questionnaires. We also examined the blood chemistry (including plasma glucose, HbA1c, insulin level, and serum levels of creatinine) during a fasting state. The HbAlc level was measured using high-performance liquid chromatography and was expressed as the international standard value, i.e., HbA1c (1.02 × Japan Diabetes Society [JDS (%)] + 0.25%), as defined by the JDS [27]. The HOMA-IR was calculated as previously reported [28].

### Adiponectin measurement

Among the subjects, three adiponectin isoforms (HMW, MMW, and LMW) were measured using an enzyme-linked immunosorbent assay (ELISA) (SEKISUI Medical Co. Ltd., Japan) [12, 20, 29]. This ELISA had a dynamic range of 0.075–4.8 ng/mL. The intra-assay coefficients of variation values were 5.3% (total adiponectin), 4.1% (MMW+HMW), and 3.3% (HMW).

### Covariates

Age, sex, BMI and serum creatinine were evaluated as covariates because these factors have been shown to affect either the diabetic status or the level of total adiponectin and the three adiponectin isoforms (HMW, MMW, and LMW), as previously reported [20, 30–33]; we confirmed this finding in the 1^st^ group (Table 3).

**Table 3:** Correlations between various adiponectin-related parameters and clinical parameters in the 1^st^ group.

Variables	age		sex(women = 0, men = 1)		BMI		S-Cre	
	r	*P*	r	*P*	r	*P*	r	*P*
Total-Adiponectin^a^	0.308	<0.0001	-0.351	<0.0001	-0.317	<0.0001	-0.046	0.202
HMW-Adiponectin^a^	0.275	<0.0001	-0.351	<0.0001	-0.302	<0.0001	-0.081	<0.05
MMW-Adiponectin^a^	0.280	<0.0001	-0.289	<0.0001	-0.232	<0.0001	-0.025	0.494
LMW-Adiponectin^a^	0.246	<0.0001	-0.181	<0.0001	-0.235	<0.0001	0.078	<0.05
HMW/Total	0.184	<0.0001	-0.328	<0.0001	-0.25	<0.0001	-0.149	<0.0001
MMW/Total	-0.038	0.289	0.087	<0.05	0.142	<0.0001	0.021	0.559
LMW/Total	-0.186	<0.0001	0.322	<0.0001	0.202	<0.0001	0.157	<0.0001

r, versus various adiponectin-related parameters, Pearson’s correlation coefficient

BMI, body mass index; S-Cre, serum levels of creatinine; Total, total adiponectin; HMW, high molecular weight adiponectin; MMW, middle molecular weight adiponectin; LMW, low molecular weight adiponectin

^a^ Parameters were transformed logarithmically before analysis.

### Statistical analysis

Categorical data were expressed as a percentage, while continuous data values were expressed as the mean ± SD. The statistical analyses were performed using JMP for Windows, Version 10.0 (SAS Institute, Cary, NC, USA). The normality of the distributions was checked using the skewed score, and variables with skewed distributions were logarithmically (naturally) transformed in subsequent analyses. Differences in continuous variables for clinical features and the adiponectin-related parameters between T2D subjects and controls were examined using the Student *t*-test and a multiple logistic regression analysis after adjustments for related co-variables (Tables 1 and 4). The relationships between the adiponectin-related parameters and the clinical parameters were investigated using a simple regression analysis in the 1^st^ group (Table 3). The odds ratios (ORs) for T2D according to the adiponectin-related parameters were calculated using a logistic regression analysis with confounding factors (Table 5). The association of the various adiponectin-related parameters with the HOMA-IR were examined by calculating the β values using a multiple linear regression analysis with adjustments for related co-variables (Table 6). Results with *P* values <0.05 were considered statistically significant.

**Table 4:** Comparison of the various adiponectin-related parameters between diabetic subjects and control subjects in the 1^st^ group.

	Diabetes	Control	*P* value	*P* values* Multivariate
Total-Adiponectin (μg/mL)^a^	5.12 ± 2.74	7.88 ± 3.73	<0.001	<0.001
HMW-Adiponectin (μg/mL)^a^	2.25 ± 1.73	3.84 ± 2.53	<0.001	<0.001
MMW-Adiponectin (μg/mL)^a^	1.15 ± 0.62	1.70 ± 0.90	<0.001	<0.001
LMW-Adiponectin (μg/mL)^a^	1.72 ± 0.66	2.34 ± 1.06	<0.001	<0.001
HMW/Total	0.39 ± 0.13	0.45 ± 0.13	<0.001	<0.01
MMW/Total	0.23 ± 0.06	0.23 ± 0.08	0.263	0.847
LMW/Total	0.38 ± 0.11	0.33 ± 0.11	<0.001	<0.01

Total, total adiponectin; HMW, high molecular weight; MMW, middle molecular weight; LMW, low molecular weight

*Multivariate *P* values were adjusted for sex, age, log BMI, and serum creatinine level

^a^ Parameters were transformed logarithmically when compared using the Student *t*-test and a multiple logistic regression analysis.

**Table 5:** Odds ratios of adiponectin-related parameters for diabetes in the 1^st^ group.

Index-Level	Odds ratio	95%CI	*P* value
Total-Adiponectin (μg/mL)^a^	0.25	(0.17–0.35)	<0.0001
HMW-Adiponectin (μg/mL)^a^	0.53	(0.42–0.65)	<0.0001
MMW-Adiponectin (μg/mL)^a^	0.34	(0.24–0.47)	<0.0001
LMW-Adiponectin (μg/mL)^a^	0.24	(0.16–0.36)	<0.0001
Index-Ratio	Odds ratio	95%CI	*P* value
HMW/Total	0.20	(0.06–0.69)	<0.01
MMW/Total	0.78	(0.08–7.64)	0.83
LMW/Total	8.64	(2.13–35.74)	<0.01

^a^ Parameters were transformed logarithmically before analysis.

Total, total adiponectin; HMW, high molecular weight; MMW, middle molecular weight; LMW, low molecular weight

Multivariate *P* values were adjusted for sex, age, log BMI, and serum creatinine level

**Table 6:** Association of adiponectin-related parameters with HOMA-IR in the 2^nd^ group.

Index-Level	β†	SE	*P* value
Total-Adiponectin (μg/mL)^a^	-0.34	0.06	<0.0001
HMW-Adiponectin (μg/mL)^a^	-0.19	0.04	<0.0001
MMW-Adiponectin (μg/mL)^a^	-0.22	0.05	<0.0001
LMW-Adiponectin (μg/mL)^a^	-0.18	0.06	<0.01
Index-Ratio	β	SE	*P* value
HMW/Total	-0.83	0.21	<0.001
MMW/Total	0.41	0.36	0.255
LMW/Total	0.89	0.24	<0.001

^a^ Parameters were transformed logarithmically before analysis.

Total, total adiponectin; HMW, high molecular weight; MMW, middle molecular weight; LMW, low molecular weight

^†^ regression coefficient adjusted for age, sex, log BMI, serum creatinine level, and the presence of diabetes mellitus.

## Results

In the 1^st^ group, the mean BMI and the mean ages in the T2D group were significantly higher and younger, respectively, than those in the non-diabetic controls (current BMI: 24.2 ± 3.8 kg/m^2^ vs. 23.0 ± 3.0 kg/m^2^, *P* < 0.001; mean age: 63.9 ± 10.9 years vs. 67.4 ± 8.0 years, *P* < 0.0001) (Table 1). The percentages of subjects with a male gender and who had received medication for hypertension and hyperlipidemia were significantly higher among the T2D subjects than among the controls (percentage of male gender: 65.2% vs. 41.7%, *P* < 0.0001; percentage of subjects who took antihypertensive drugs: 55.6% vs. 36.1%, *P* < 0.0001; percentage of subjects who took lipid-lowering drugs: 46.4% vs. 21.9%, *P* < 0.0001) (Table 1). The mean duration of diabetes and the percentage of subjects who received diabetic medication including oral hypoglycemic agents and/or insulin therapy were 13.4 ± 8.8 years and 84.3%, respectively (Table 1). Since we recruited diabetic subjects between January 2008 and December 2009, none of the subjects were taking dipeptidyl peptidase-4 inhibitor, glucagon-like peptide-1 receptor agonist, or sodium glucose co-transporter 2 inhibitor (Table 1).

Next, we compared the various adiponectin-related parameters between diabetic subjects and controls in the 1^st^ group. We found that the levels of HMW, MMW, and LMW adiponectin (A) were significantly lower among the diabetic subjects than among the controls when examined with and without adjustments for related co-variables (HMW-A: 2.3 ± 1.7 μg/mL vs. 3.8 ± 2.5 μg/mL, *P* < 0.0001; MMW-A: 1.1 ± 0.6 μg/mL vs. 1.7 ± 0.9 μg/mL, *P* < 0.0001; LMW-A: 1.7 ± 0.7 μg/mL vs. 2.3 ± 1.1 μg/mL; *P* < 0.0001) (Table 4). Regarding the ratios of each adiponectin isoform to the total adiponectin value for the diabetic subjects, the HMW/total (H/T) ratio was significantly lower while the LMW/total (L/T) ratio was significantly higher compared with the ratios among the controls, with and without adjustments for confounding factors (H/T ratio: 0.39 ± 0.13 vs. 0.45 ± 0.13, *P* < 0.01; L/T ratio: 0.38 ± 0.11 vs. 0.33 ± 0.11, *P* < 0.01) (Table 4). Next, we investigated the associations of the various adiponectin-related parameters with T2D using a logistic regression analysis with adjustments for confounding factors (Table 5). The total, HMW-A, MMW-A, and LMW-A levels and the H/T ratio were significantly and negatively associated with diabetes (HMW-A: OR = 0.53 [95% CI, 0.42–0.65], *P* = 6.0 × 10^−10^; MMW-A: OR = 0.34 [95% CI, 0.24–0.47], *P* = 3.5 × 10^−12^; LMW-A: OR = 0.24 [95% CI, 0.16–0.36], *P* = 1.3 × 10^−12^; H/T ratio: OR = 0.20 [95% CI, 0.06–0.69], *P* < 0.01), while the L/T ratio was significantly and positively associated with diabetes (OR = 8.64 [95% CI, 2.13–35.74], *P* < 0.01) (Table 5).

We next investigated the associations of the various adiponectin-related parameters with the HOMA-IR using a multiple regression analysis with adjustments for confounding factors in the 2^nd^ group (Table 6). Like the association with diabetes, the HMW-A, MMW-A, and LMW-A levels and the H/T ratio were significantly and negatively associated with the HOMA-IR (β ln- HOMA-IR for HMW-A = -0.19, SE = 0.04, *P* = 1.9 × 10^−7^; β ln- HOMA-IR for MMW-A = -0.22, SE = 0.05, *P* = 2.8 × 10^−5^; β ln- HOMA-IR for LMW-A = -0.18, SE = 0.06, *P* < 0.01; β ln- HOMA-IR for H/T ratio = -0.83, SE = 0.21, *P* < 0.001), while the L/T ratio was significantly and positively associated with the HOMA-IR (β ln- HOMA-IR for L/T ratio = 0.89, SE = 0.24, *P* < 0.001) (Table 6). The MMW/total ratio was not associated with diabetes or the HOMA-IR (Tables 5 and 6).

## Discussion

In this study, we found that in the 1^st^ and 2^nd^ groups, the levels of all the adiponectin isoforms and the HMW/total ratio were significantly lower among the diabetic subjects than among the controls and were negatively associated with T2D and the HOMA-IR. On the contrary, the LMW/total ratio was significantly higher among the diabetic subjects and was positively associated with T2D and the HOMA-IR. Meanwhile, the MMW/total ratio was not associated with T2D or the HOMA-IR.

Until now, although several studies have investigated the diabetes-adiponectin association [6–10, 13, 15, 34–38], most of these studies have examined only the level of total and HMW adiponectin and have not evaluated the association between diabetes and other adiponectin isoforms, such as MMW and LMW. To the best of our knowledge, although a few groups have recently investigated the associations between the levels of　MMW and LMW adiponectin and the presence of diabetes [17–19], their conclusions were controversial, as mentioned in the Introduction section. In addition, among the three adiponectin isoforms, although a lower HMW/total ratio has been reported to be associated with insulin resistance and T2D [13, 15, 16, 39], indicating that the HMW/total ratio was decreased in T2D subjects, to the best of our knowledge, no studies have examined how the ratio of other adiponectin isoforms to the total adiponectin level might differ in subjects with T2D. Therefore, the present study is the first to investigate the association of the MMW/total and LMW/total ratios with insulin resistance and diabetes and to demonstrate that an increased LMW/total ratio is significantly and positively associated with insulin resistance and diabetes, suggesting that, in contrast to the reduction in the ratio of HMW/total, the increased LMW/total ratio was associated with T2D through its relation to insulin resistance. These findings suggest that an evaluation of the LMW/total ratio may be useful for predicting the onset of diabetes mellitus in non-diabetic subjects. In addition, the measurement of the LMW/total ratio in diabetic subjects may be useful for evaluating the etiology of their diabetes and for selecting an appropriate therapy (e.g., insulin-sensitizing drugs for diabetic subjects with a relatively high LMW/total ratio).

Although the exact mechanism explaining the relationship between a high LMW/total ratio and insulin resistance remains to be determined, the following two theories are possible. The first involves a competitive relationship between the HMW and LMW fractions, as previously reported by Bouskila et al. [14, 40]. They speculated that LMW adiponectin might act as an antagonist to adiponectin activity at the level of the receptor by preventing HMW adiponectin-mediated receptor clustering and signaling activation. In addition, they also speculated that LMW adiponectin might also be a competitive inhibitor of an upstream activator of a ligand, such as a reductase and/or a protease, with the ability to cleaves the full-length HMW adiponectin proteolytically and to produce a truncated adiponectin with the ability to activate downstream signaling cascades, such as AMP-activated protein kinase. From these speculations, the higher ratio of LMW adiponectin-induced insulin resistance might be secondary to its inhibitory effect on the amelioration of insulin sensitivity induced by HMW adiponectin. Another possibility might arise from the inverse relationship between the level of total adiponectin and the LMW/total ratio, as demonstrated in the previous papers [20, 41]. We and another group have reported that a lower level of total adiponectin can cause a higher LMW/total ratio in cross-sectional studies [20, 41]. Therefore, the higher LMW/total ratio found in diabetic subjects might depend on a lower total adiponectin level, which could induce insulin resistance. However, in these previous reports, we and another group showed that a lower level of total adiponectin could also cause a high MMW/total ratio. In the present study, however, we did not observe any difference in the MMW/total ratio between diabetic subjects and control subjects, although a lower level of total adiponectin was observed in the diabetic subjects (Table 4). Therefore, we speculated that the higher LMW/total ratio might be associated with insulin resistance through a different mechanism from that related with the lower total adiponectin level.

MMW and LMW adiponectin are reported to be able to go through the blood-brain barrier and suppress energy metabolism and promote appetite by influencing the hypothalamus [42]. However, data concerning the physiological role of LMW adiponectin in glucose metabolism remains extremely rare. Pajvani et al. examined the effect of LMW and HMW adiponectin on the serum glucose level in adiponectin-knockout mice and demonstrated that LMW adiponectin did not affect the serum glucose level, whereas HMW adiponectin dose-dependently reduced the level [14]. Therefore, the basic mechanism for the association between LMW adiponectin and glucose metabolism is not determined.

The major strength of this study is that it compares not only the total and HMW adiponectin levels, but also the levels of other isoforms of adiponectin and their relative ratios between diabetic and control subjects, revealing a significant association between an increased LMW/total ratio and insulin resistance and diabetes. These findings are novel. Nevertheless, this study have some limitations. First, regarding the analysis of the association between diabetes and adiponectin, we could not examine the association in the age-, sex- and BMI-matched subjects between the diabetes and control groups. The mean age and percentage of subjects with a female gender were significantly higher and the BMI was lower for the control subjects than for the diabetic subjects. Since aging, a lower BMI, and a female gender have all been reported to increase the total adiponectin level and the levels of its three isoforms in previous reports [20, 32, 33] and the present study (Table 3), these differences in characteristics between the diabetic subjects and controls might have affected our findings. However, in the present study, we evaluated the relationship between diabetes and adiponectin using not only the Student *t*-test, but also a multiple logistic regression analysis with adjustments for related co-variables including age, sex and BMI, and a significant relationship was observed. Second, we could not obtain any information regarding the alcohol drinking and smoking statuses of the subjects, which might have affected the levels of total adiponectin and its three isoforms [17]. The lack of this information might have affected our findings. However, in a previous report conducted in Japan that investigated the associations of the three adiponectin isoforms with diabetes among elderly individuals, the authors concluded that the LMW adiponectin level was inversely related with diabetes with adjustment for confounding factors including the smoking and alcohol drinking statuses [17]. This conclusion was consistent with our findings in the present study, and we think that the absence of this information likely had only a minimal effect on our conclusion. Third, the modification of adiponectin-related parameters by the administration of various drugs should also be taken into consideration. In the present study, about 80% of the diabetic subjects were taking diabetic medication, and the percentages of subjects who took lipid-lowering agents and antihypertensive drugs were higher among the diabetic subjects than among the controls. In addition to TZDs [23, 24], diabetic medication (including insulin therapy, sulfonylureas, metformin, α-glucosidase inhibitors and glinide), antihypertensive agent (angiotensin II type 1 receptor blockers, etc.), and lipid lowering drug (fibrates) might also affect the serum total adiponectin level [43]. However, since these agents have been reported to possibly increase the serum total adiponectin level, but not to decrease the level [43–48], we think that the modification of the serum adiponectin level by various drugs might not have affected our findings regarding the reductions in the total, HMW, MMW and LMW adiponectin levels in diabetic subjects. Fourth, to analyze the association of the three adiponectin isoforms with diabetes, we randomly selected diabetic and control subjects from among the participants of a previously reported cohort study [22], which was not a population-based cohort. Since differences in the background characteristics of the diabetic and control subjects in the present study were observed (Table 1), a selection bias may exist. Fifth, to measure the three adiponectin isoforms, we used the SEKISUI ELISA kit (SEKISUI Medical Co. Ltd., Japan)[12, 20, 29], as mentioned in the Materials and Methods section. In this kit, the LMW adiponectin values are calculated by subtracting the HMW and MMW adiponectin values from the total adiponectin value. Therefore, with this kit, it can be expected that unless the MMW/total ratio changes, the LMW/total ratio is higher in situations where the HMW/total ratios are lower. Therefore, the higher LMW/total adiponectin ratio in diabetic patients observed in this study may simply reflect the lower HMW/total adiponectin ratio in these patients. However, in this study, although we observed no difference in the MMW/total ratio between the diabetic and control subjects overall, the MMW/total ratio may change in individual subjects if they also have conditions such as asthma[20], endometrial cancer[49] or other unknown diseases affecting the MMW adiponectin level. In this case, an accurate evaluation of the associations between the LMW/total ratio and diabetes and insulin resistance might not be possible using this kit. Therefore, a kit that is able to measure the LMW adiponectin concentration directly without being affected by the HMW and/or MMW adiponectin levels is needed in the future, such as an ELISA assay for measuring the level of HMW adiponectin (Fujirebio, Tokyo, Japan)[50]. With such a development, we would be able to evaluate the association between the LMW/total ratio and diabetes more accurately. Finally, since this study was a cross-sectional analysis, we could not establish a temporal relationships between a higher LMW/TA ratio and an increased risk of T2D.

In conclusion, we have shown that the increased LMW/total ratio was significantly and positively associated with T2D and insulin resistance, in contrast to the reduction in the levels of all the adiponectin isoforms and the ratio of HMW/total. However, this study was a cross-sectional analysis performed in a relatively small sample size. A prospective study involving a larger number of subjects and basic research about the association between LMW adiponectin and diabetes are needed to clarify our findings in the present study.
